# Quantification of Root Growth Patterns From the Soil Perspective via Root Distance Models

**DOI:** 10.3389/fpls.2018.01084

**Published:** 2018-07-24

**Authors:** Steffen Schlüter, Sebastian R. G. A. Blaser, Matthias Weber, Volker Schmidt, Doris Vetterlein

**Affiliations:** ^1^Department of Soil System Science, Helmholtz-Centre for Environmental Research – UFZ, Halle, Germany; ^2^Institute of Stochastics, Ulm University, Ulm, Germany; ^3^Soil Science, Martin-Luther-University Halle-Wittenberg, Halle, Germany

**Keywords:** x-ray tomography, euclidean distance, root system architecture, time-lapse imaging, parametric model

## Abstract

The rhizosphere, the fraction of soil altered by plant roots, is a dynamic domain that rapidly changes during plant growth. Traditional approaches to quantify root growth patterns are very limited in estimating this transient extent of the rhizosphere. In this paper we advocate the analysis of root growth patterns from the soil perspective. This change of perspective addresses more directly how certain root system architectures facilitate the exploration of soil. For the first time, we propose a parsimonious root distance model with only four parameters which is able to describe root growth patterns throughout all stages in the first 3 weeks of growth of *Vicia faba* measured with X-ray computed tomography. From these models, which are fitted to the frequency distribution of root distances in soil, it is possible to estimate the rhizosphere volume, i.e., the volume fraction of soil explored by roots, and adapt it to specific interaction distances for water uptake, rhizodeposition, etc. Through 3D time-lapse imaging and image registration it is possible to estimate root age dependent rhizosphere volumes, i.e., volumes specific for certain root age classes. These root distance models are a useful abstraction of complex root growth patterns that provide complementary information on root system architecture unaddressed by traditional root system analysis, which is helpful to constrain dynamic root growth models to achieve more realistic results.

## Introduction

Root-soil interactions are an essential part of global matter cycles as all water and nutrients taken up by the plant have to be transported through the rhizosphere (York et al., 2016). Roots have to fulfill a range of different functions at the same time, resulting in the plasticity of the root system, with individual root segments changing their function during ontogeny (Vetterlein and Doussan, 2016; Morris et al., 2017). The consortium of root segments comprising the root system can thus adapt to heterogeneity in resource availability and demand in time and space (Carminati and Vetterlein, 2012). The actual root system architecture is both a manifestation of genetic predisposition and environmental factors (De Smet et al., 2012). There is a genotype specific regulation of root development (Atkinson et al., 2014). However, this program is modified by soil traits like bulk density, soil structure, water distribution or nutrient supply (Drew, 1975; Passioura, 1991; Robinson, 1994; Malamy, 2005; Hodge et al., 2009; Smith and De Smet, 2012; Giehl and von Wiren, 2014).

The rhizosphere, i.e., the zone of soil modified by the roots, is closely related to root system architecture. The spatial arrangement of root segments determines the fraction of the soil volume directly altered by roots with respect to a specific process. An even distribution of root segments may be advantageous, and cost effective in terms of carbon investment as well as for the acquisition of resources that have a high fluctuation over time like water. However, clustering of roots may be beneficial for resource acquisition that requires alteration of biochemical properties (Ho et al., 2005; Lynch and Ho, 2005).

Methods to quantify root system architecture—the three-dimensional distribution of the root system from a single plant within the soil volume—have so far mainly focused on the plant perspective (Danjon and Reubens, 2008; Iyer-Pascuzzi et al., 2010; Clark et al., 2011; Flavel et al., 2017). Traditionally, destructive sampling is carried out, separating the roots from soil by washing and detecting roots visually (Tennant, 1975) or by semi-automatic detection with a flat scanner and analysis with WinRHIZO (Regent Instruments, Canada). Results are presented as root length density, root surface or root volume distributed over a certain sampling depth, occasionally, specified for certain root diameter classes. Alternatively root system architecture in the field has been described by tedious and only semi-quantitative root profile methods and drawings as in Kutschera (1960).

The rise of non-invasive imaging methods like X-ray computed tomography (X-ray CT) and magnetic resonance imaging (MRI) has enabled the analysis of undisturbed root system architecture (Helliwell et al., 2013; Schulz et al., 2013). This has brought about additional insights into root networks like branching patterns (Flavel et al., 2017), root-soil contact (Carminati et al., 2012; Schmidt et al., 2012) and root growth response to localized application of e.g., phosphorus (Flavel et al., 2014). In addition, it enables repeated sampling to analyze root growth dynamics (Koebernick et al., 2014, 2015; Helliwell et al., 2017). However, these approaches focus on the plant perspective and are not able to describe all spatial aspects of root-soil interactions. Complementary information on root growth patterns is provided instead by a shift toward the soil perspective. That is, the root growth patterns are not characterized solely based on root traits, but based on consequences that these patterns have for the exploration of soil. For any soil voxel, the distance to the closest root voxel can be determined by employing the so-called Euclidean distance transform on segmented 3D root images. The concept has been suggested by van Noordwijk et al. (1993), however, at the time they could only apply it to a stack of 2D slices from resin embedded samples and calculations were very tedious. This might explain why the concept has not been adopted more widely, despite the fact that numerous studies have shown that alterations of soil properties by the root in the rhizosphere extend to a distance which is specific for the process in question (Hinsinger et al., 2009). We suggest to use distance maps not only as a tool to approximate travel distances in radial transport to and from the root, but also as a genuine alternative to describe root system architecture. To our knowledge the only study in this regard was reported by Koebernick et al. (2014). They showed that the frequency distribution of root distances integrated over all soil voxels, from now on denoted as root distance histogram (RDH), typically exhibits a shift from long to short root distances as the root network develops and explores the soil.

We will compare the information which can be derived from this new approach, to the classical half-mean distance parameter. Half-mean distance is used as an approximation in many modeling approaches, when real spatial information is missing. We show that the average distance to root segments estimated from an RDH can be linked to the root length density *R*_*L*_ [$\frac{L}{L^{3}}$, e.g., cm/cm3] through the theoretical half-mean distance *HMD* [*L*]

(1) $$HMD={\left(\pi R_{L}\right)}^{\frac{-1}{2}}$$

a formula which has been derived for equidistant ensembles of cylindrical roots (Gardner, 1960; Newman, 1969; de Parseval et al., 2017). However, it is unclear whether this relationship still holds for a natural, more complex root system, since experimental studies on such a comparison are lacking.

The objective of the present paper is to advocate the use of root distance histograms as complementary information on root system architecture, which remains unaddressed by traditional metrics focused on root density and morphology. We will show that root distance histograms evolve in a very regular manner, which can be predicted by means of a simple model with only four parameters. The experimental dataset to calibrate and validate the model stems from a recent study about radiation effects on early root development in *Vicia faba* (Blaser et al., 2018).

## Theoretical background

**Figure 1:**
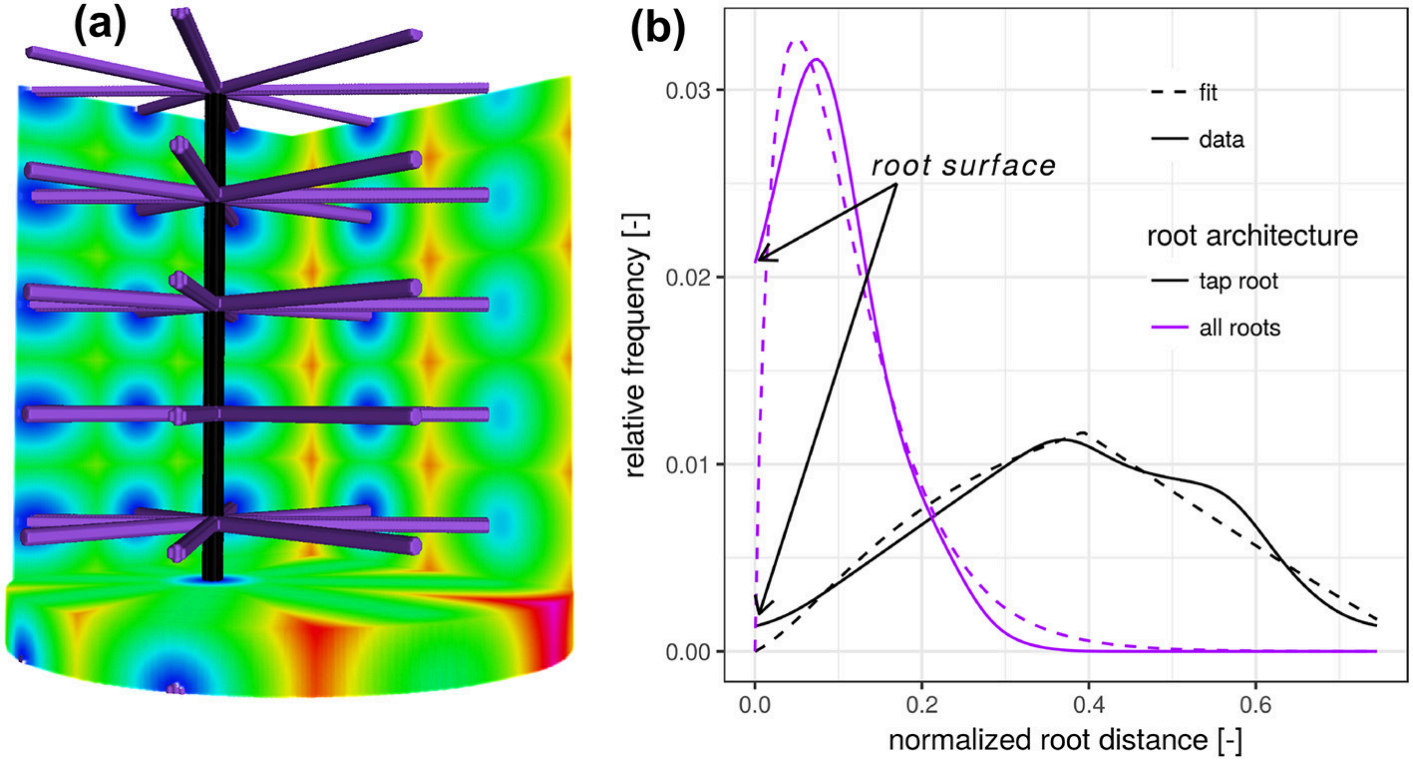
**(a)** Synthetic test image of a vertical tap root only (black) and a fully developed root architecture including the tap root and lateral roots at various depths (purple). **(b)** The root distance histogram (RDH) of the single, vertical tap root follows a triangular distribution, whereas the addition of lateral roots changes the RDH toward a Gamma distribution.

To better understand the nature of the distribution of the Euclidean distance from a randomly chosen point in soil to the nearest root voxel, we take a closer look at two synthetic test cases (Figure 1). For both test cases considered, we fix a cylindrical region of interest (ROI) in line with sample geometries used in X-ray tomography analysis. First, we create one vertical root, typical for the tap root of a dicotyledonous plant (black). We assume that the horizontal coordinates of the root are not aligned with the center of the soil column, as it is frequently observed in real pot experiments. Calculating the Euclidean distance transform inside the cylinder leads to a roughly triangular-shaped distance distribution, whose probability density function is given by

(2) $$f_{p}^{\Delta }\left(d\right)=\{\begin{array}{c} \frac{d}{p^{2}},\;if\;0\leq d\leq p,\\ \frac{2p-d}{p^{2}},\;if\;p<d\leq 2p,\\ 0,\;if\;d>2p, \end{array}$$

where *d* [*L*] denotes the radial distance from the vertical root. This distribution has only one parameter, *p* [*L*], reflecting the distance between the vertical root and the wall. The linearity in the left slope follows from the linear relationship between radial distance and perimeter. The exact slope depends on the ROI diameter and the exact position of the root. The tailing toward larger distances is a result of the random horizontal root position. For young tap roots the right tailing can also be caused by incomplete vertical exploration of the soil, which contributes larger distances to the RDH that originate from the unexplored lower ROI layer (not shown).

The addition of lateral roots (purple) to the tap root changes the RDH toward a Gamma distribution with density

(3) $$f_{k,\theta }^{\Gamma }\left(d\right)=d^{k-1}\frac{\exp\left(\frac{-d}{\theta }\right)}{\theta^{k}\Gamma \left(k\right)},\;for\;all\;d\geq 0$$

with distance *d* [*L*], scaling parameter θ [*L*] and dimensionless shape parameter *k*. Here, Γ denotes the Gamma function, i.e., $\Gamma \left(k\right)=\int_{0}^{\infty }x^{k-1}e^{-x}dx$. The shape parameter *k* has two special cases, the exponential distribution for *k* = 1 and the Gaussian distribution for *k* = ∞. While the scaling parameter θ is likely to reflect the general exploration of soil by roots, the shape parameter *k* is more likely to reflect the balance between the frequency of minimal distances and most frequent distances, depicted in blue and green in Figure 1a. In this synthetic test case the expected Euclidean root distance $\langle f_{k,\theta }^{\Gamma }\left(d\right)\rangle =k\theta$ is mainly governed by the vertical separation distance between laterals. In this particular example the variance $var\left(f_{k,\theta }^{\Gamma }\left(d\right)\right)=k\theta^{2}$ is mainly governed by the ratio between sample diameter and the vertical separation distance between laterals. The intercept with the *y*-axis at zero distance is increased because there are more soil voxels located directly at the root surface. The combination of Equations (2) and (3) leads to the proposed root distance model, the so-called mixed triangular-gamma distribution with density

(4) $$f_{c,k,\theta ,p}\left(d\right)=cf_{k,\theta }^{\Gamma }\left(d\right)+\left(1-c\right)f_{p}^{\Delta }\left(d\right),\;for\;all\;x\geq 0$$

which has one additional parameter, *c* ∈ [0, 1], the dimensionless weighting factor for linear mixing of both densities. This mixed triangular-gamma distribution will be used to model intermediate structural scenarios between a vertical tap root only and a fully developed root architecture including the tap root and lateral roots at various depths.

## Materials and methods

**Figure 2:**
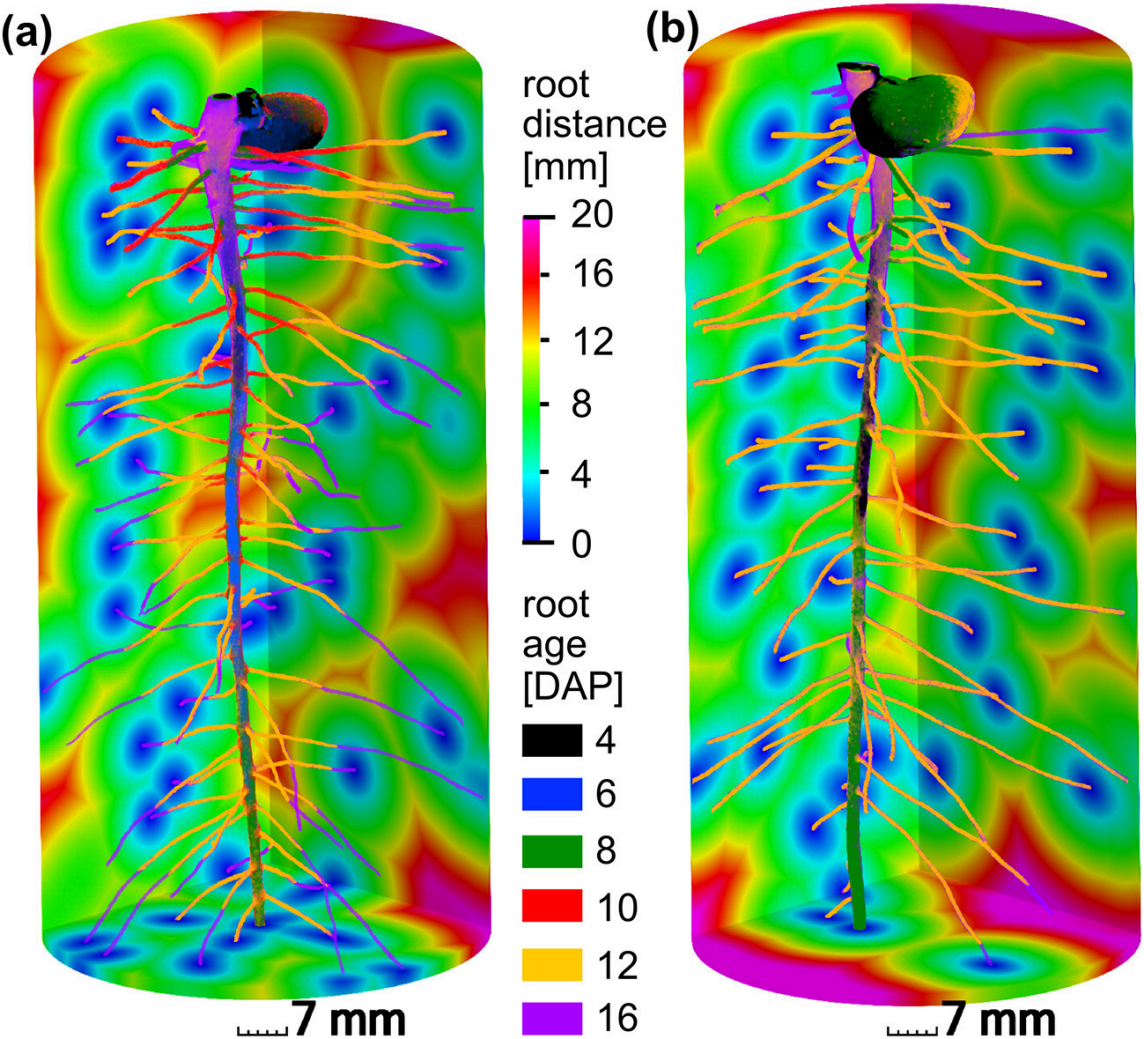
Different root growth dynamics of *Vicia faba* in two radiation treatments: **(a)** frequent scanning (FS) every second day exposed to an estimated total dose of 7.8 Gy, **(b)** moderate scanning (MS) every fourth day exposed to an estimated total dose of 4.2 Gy. Accordingly, time step (6 DAP, blue) and (10 DAP, red) are only available for frequent scanning **(a)**. The main difference between both treatments is the slower growth of laterals around 12 days after planting (DAP) in the FS treatment. The root distances in the soil are depicted for the final time step at 16 DAP.

The present paper is based on the data obtained in a study on radiation effects on early root development in faba bean (Blaser et al., 2018). The experimental setup is briefly summarized here. *Vicia faba* plants (L., cv. “Fuego”) were grown in cylindrical columns (250 mm height, 35 mm radius, 5 mm wall thickness) filled with sieved (<2 mm) silty clay loam with a bulk density of 1.2 g/cm3 and a constant volumetric water content of 27%. Root growth during the first 17 days after planting (DAP) was detected via X-ray CT. Two treatments (with 5 biological replicates each) were considered for this study, differing in frequency of X-ray CT scanning. In the high radiation treatments, from now on denoted as frequent scanning (FS), samples were scanned every second day and exposed to an estimated, total radiation of 7.8 Gy. In the low radiation treatment, from now on denoted as moderate scanning (MS), samples were only scanned every fourth day resulting in an estimated total dose of 4.2 Gy. Doses were calculated with the Rad Pro Calculator Version 3.26 (McGinnis, 2009). For both treatments the first application of X-ray CT was performed at 4 DAP. The X-ray CT images were filtered with Gaussian smoothing and segmented with semi-automated region growing. Registration of segmented images of consecutive time steps of the same sample was performed in order to achieve the best visualization of growth dynamics (Figure 2) and to enable subsequent analysis of distances related to root age. A root age image was computed using simple image arithmetic, i.e., a gray value represents the time step, when a voxel was assigned to the root class for the first time. By means of a skeletonization algorithm the root network was analyzed with respect to total root length density and individual root length densities of tap roots and lateral roots. Detailed information about the growth conditions, X-ray CT scan settings and all image processing steps can be retrieved from Blaser et al. (2018). For each treatment, examples of a root network with age information and root distances in the soil matrix are depicted in Figure 2.

## Results

### Root distance histograms

**Figure 3:**
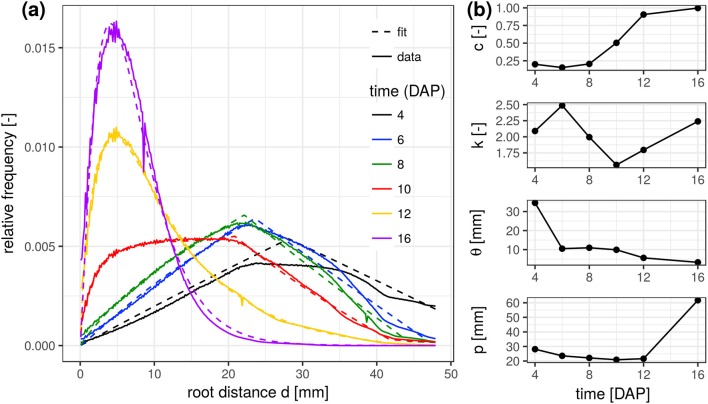
**(a)** Root distance histograms for all time steps in the FS treatment including the fitted mixed triangular-gamma models (Equation 4). **(b)** Time series of the four parameters of the mixed triangular-gamma model: *c*, weighting factor; *k*, shape parameter; θ, scaling parameter; *p*, tap root-wall distance.

The experimental root distance histograms in the frequent scanning treatments exhibit a clear transition from triangular distributions (4–8 days after planting) to left skewed gamma distributions (12–16 days after planting) (Figure 3a). Data for 14 DAP are left out as it barely differs from the final state at 16 DAP. The root system at 10 DAP is in a transitional stage, during which the lateral roots are already developed in the upper part of the column but still absent at the lower part. The mixed triangular-gamma model is capable of fitting all growth stages very well. The temporal evolution of all model parameters displays some consistent trends (Figure 3b). The weighting factor *c* of the gamma distribution is increasing monotonically with the development of laterals. The scale parameter θ and the shape parameter *k* are only meaningful when *c* clearly differs from zero, i.e., *c* > 0.15. In that case, θ decreases with increasing exploration of soil by laterals. The shape parameter *k*, in turn, fluctuates around 2 during all development stages, i.e., the ratio between the volume fraction of soil voxels with minimal root distance and most frequent root distance remains rather constant. The tap root-wall distance parameter *p* of the triangular model decreases while the tap root is still expanding vertically and loses meaning as *c* approaches one.

**Figure 4:**
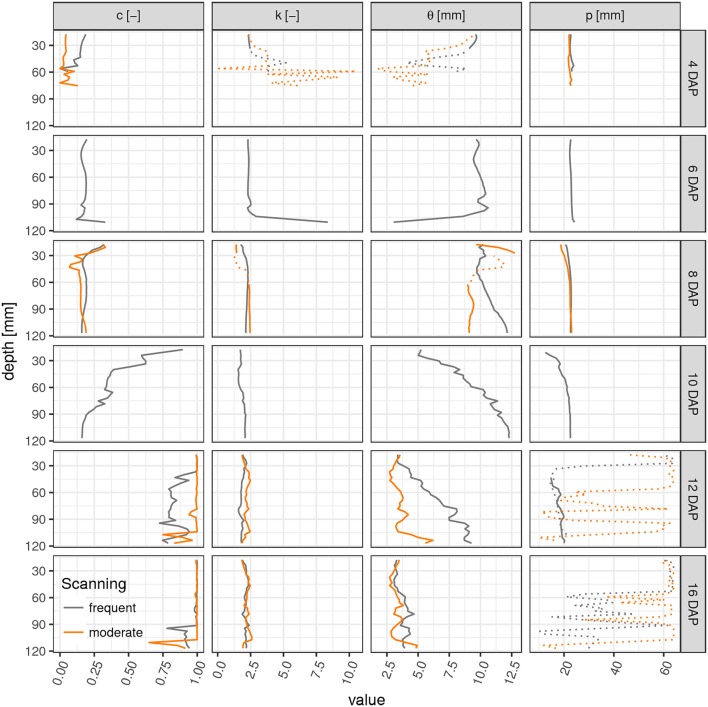
Parameter profiles (*c*, weighting factor; *k*, shape parameter; θ, scaling parameter; *p*, tap root-wall distance) of the mixed triangular-gamma model at each scan time (days after planting) in equidistant, 5 mm thick slices. Dashed lines indicate uncertain values due to imbalanced mixing of the two models in Equation (4) (*c* < 0.15 or *c* > 0.85).

Parameter profiles for each time step reveal vertical differences in the development of laterals as already discussed above for the root network at 10 DAP (Figure 4). In fact, only for this sampling date a steep transition in the weighting factor *c* exists within the soil column, i.e., from a gamma model at the top to a triangular model in the lower part. The shape parameter *k* is rather stable across all soil layers and time points except for cases when roots are generally absent (4–6 DAP, lower ROI). Apparently the value of *k* is characteristic for *Vicia faba* during all development stages, perhaps reflecting the rather constant separation distance of laterals along the tap root and the absence of secondary lateral roots in this study. The scaling parameter θ varies with depth during the transitional stages (10–12 DAP) and reflects the uneven exploration of the soil further away from the tap root. Obviously, the tap root-wall distance parameter *p* is almost constant across all depths for a constant ROI diameter and a vertically oriented tap root. The parameter *p* loses meaning when *c* > 0.85 and starts to fluctuate. Differences between FS and MS radiation treatment are also depicted in Figure 4. In line with visual inspection, the largest differences emerge at the joint sampling date 12 DAP. The MS treatment has already reached its final RDH everywhere except for the lowest layers (depth > 100 mm), whereas the FS treatment has reached this final state only at the very top (depth < 30 mm).

**Figure 5:**
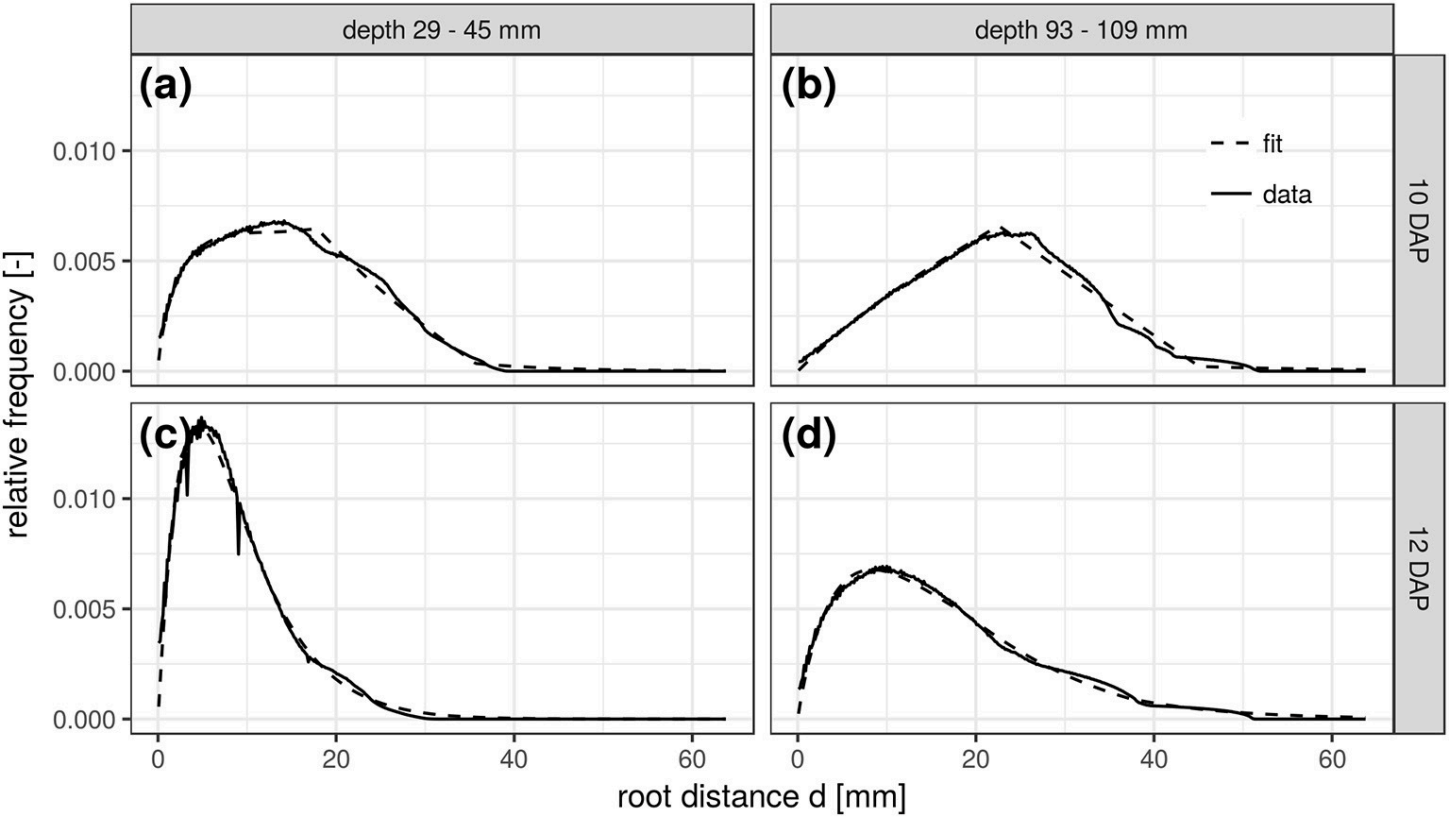
Root distance histograms in two different soil depths (**a,c**: 29–45 mm; **b,d**: 93–109 mm) at two scanning times (**a,b**: 10DAP; **c,d**: 12DAP; DAP, days after planting) of the frequent scanning (FS) radiation treatment.

This congruency of space and time is further demonstrated for two soil depths and scanning dates of the FS treatment. The RDH at 10 DAP in a shallow depth range of 29–45 mm (Figure 5a) is very similar to the RDH at 12 DAP in a larger depth range of 93–109 mm (Figure 5d). The RDH at 12 DAP in the shallow soil layer has already fully turned into a Gamma distribution (Figure 5c), a stage that is only reached at 16 DAP in the deeper soil layer (not shown). The RDH at 10 DAP in the deeper soil layer, in turn, is still dominated by the triangular distribution (Figure 5b). The mixed triangular-gamma distribution is suitable to fit the experimental RDH in all cases considered. Note, however, that this congruency of space and time is characteristic for *Vicia faba* in the radiation experiment considered in the present paper, but not necessarily the case for other plant species and growth stages beyond those studied. Potentially this is a typical phenomenon observed in tap rooted plant species but more studies are needed to proof this hypothesis.

### Rhizosphere volumes

**Figure 6:**
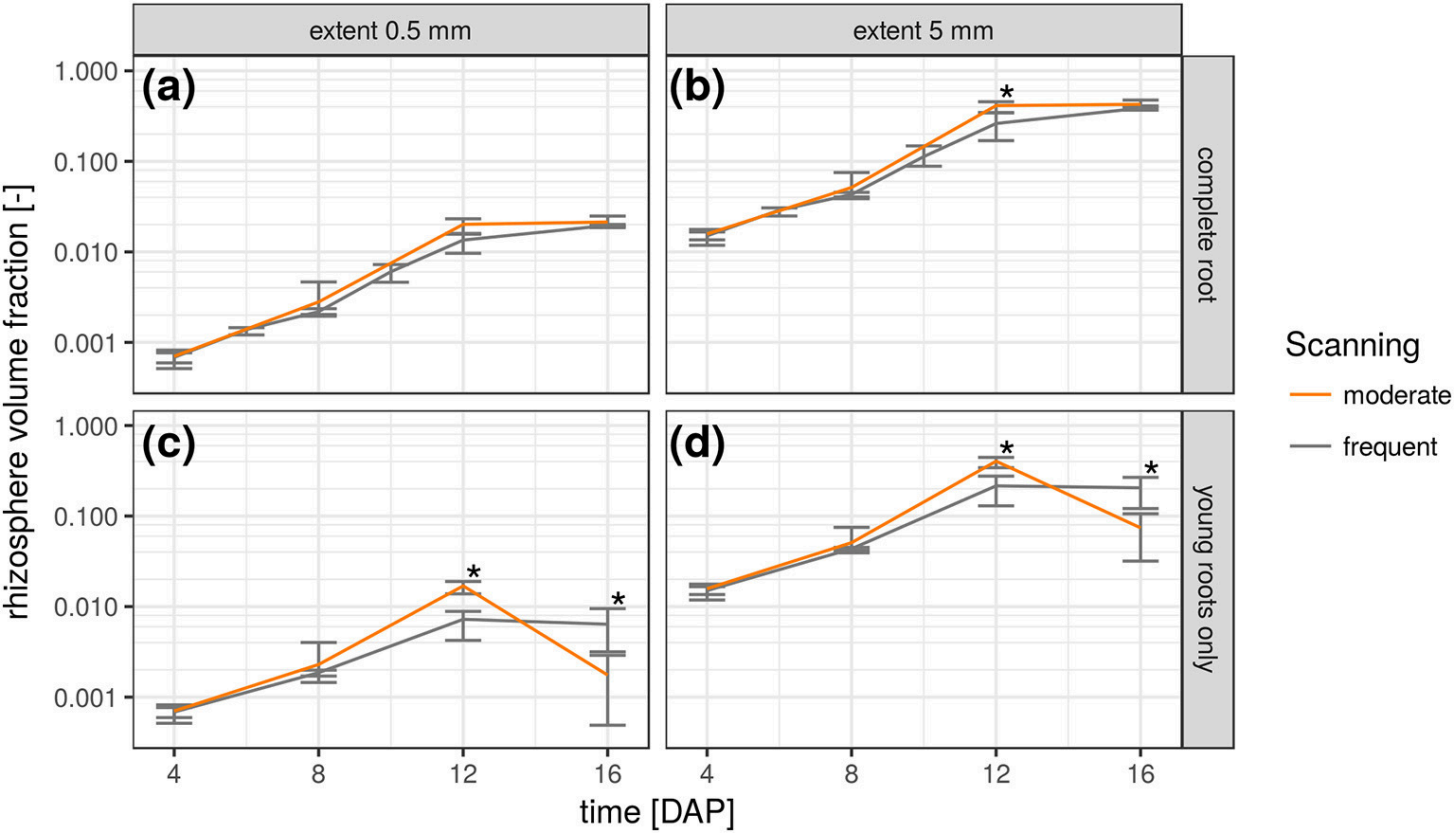
Temporal change of rhizosphere volume fraction in both radiation treatments for two hypothetical rhizosphere radii (0.5 and 5 mm) and shown separately for the complete root network **(a,b)** and only the young roots that have grown since the previous scan time **(c,d)**. Error bars refer to minimum and maximum for five biological replicates of each treatment and asterisk refers to significant differences tested at *p* < 0.05.

The relative frequency value of a certain root distance in the RDH represents the volume fraction of voxels with a given Euclidean distance to the nearest root voxel. Integrating the RDH over all distances smaller than a maximum rhizosphere extent accordingly results in the volume fraction of the rhizosphere. The extent of the rhizosphere depends on the considered process. It can be large for water depletion by root water uptake as the resulting gradient in water potential around roots drives water flow toward the roots, which stretches the zone of water depletion far into the bulk soil (Carminati et al., 2011). The capacity for this water redistribution depends on the unsaturated conductivity and water retention of the surrounding soil. The extent of the rhizosphere is much smaller for strongly adsorbed nutrients like ammonium and phosphate which are less mobile (Hinsinger et al., 2009). Substances released by the roots, like enzymes and mucilage, are also only present in a small volume of soil for their susceptibility to microbial attack (Carminati and Vetterlein, 2012). Furthermore, they are not released uniformly by the entire root network but mainly by young root segments (Vetterlein and Doussan, 2016). This variability of the rhizosphere extent in time and space can be accounted for with root age dependent RDHs as shown in Figure 6. The top row demonstrates the increase in total rhizosphere volume for both radiation treatments and two hypothetical rhizosphere extents. Approximately 2% of the soil columns are explored by the roots after 16 days in both treatments, when a rhizosphere extent of 0.5 mm is assumed. This increases to approximately 40% if the extent is enlarged to 5 mm. The differences in the growth dynamics between both radiation treatments is fully developed 12 days after planting and significant for 5 mm rhizosphere extent but has vanished at 16 DAP because root growth only occurs outside the ROI by then. This is also confirmed by the rhizosphere volume fraction of young roots only, i.e., root segments that have grown since the last scan time 4 days earlier (Figures 6c,d). Until 12 DAP, this restricted rhizosphere volume develops in line with the total rhizosphere volume. The differences between radiation treatments become significant after 12DAP. The gap in absolute values between the two rhizosphere volume fractions (young vs. total) increases with decreasing rhizosphere extent. At 16 DAP, there are less young roots in the ROI and hence their rhizosphere volume fraction decreases. Note that this decline is not an inevitable consequence of pot experiments but a manifestation of the root system architecture of *Vicia faba* in this experiment, which largely lacked the development of second order laterals that could have entered the space between the first order laterals at that growth stage.

## Discussion

### Root perspective vs. soil perspective

**Figure 7:**
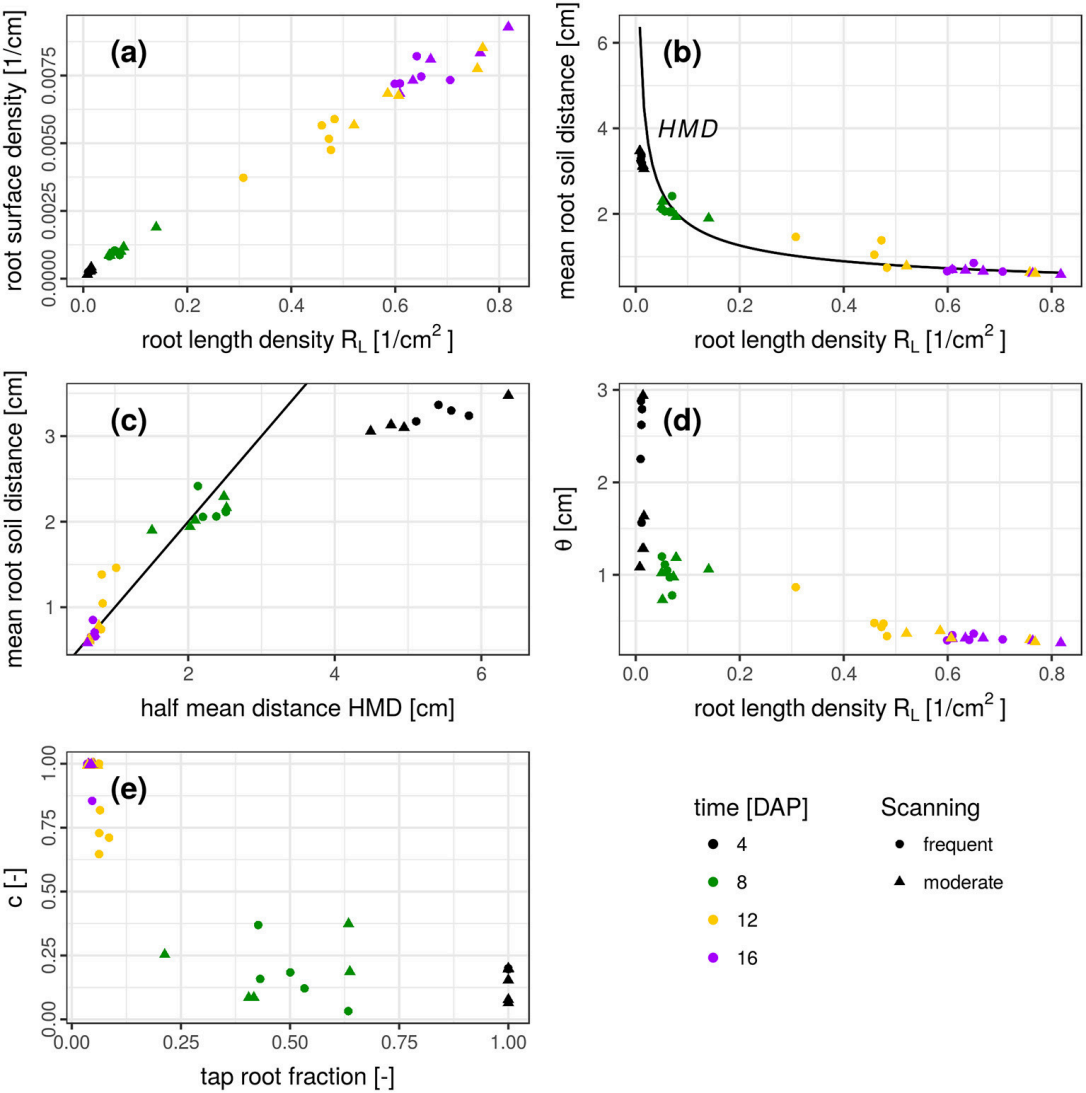
**(a-e)** Relationship between several root system traits derived from a skeleton analysis (root length density, half mean distance, tap root fraction) and root distance traits derived from mixed triangular-gamma models (relative root surface, mean root-soil distance, i.e., first central moment of the root distance histogram, and the parameters θ and *c*). Root system traits are shown on the abscissa and root distance traits on the ordinate. In **(b)**, the half mean distance derived from root length density is added as a reference for comparison (black line). In **(c)**, the 1:1 line is added.

The results presented so far describe spatial patterns in root-soil interactions from the soil perspective through distance distributions in soil and volume fractions of soil explored by roots. Traditional approaches to describe root system architectures are focused on the root perspective, e.g., by quantifying root length densities and branching patterns. We therefore discuss the question if this change in perspective provides complementary information or merely redundant information. This is assessed by comparing features of the root distance histogram with results obtained by skeletonization analysis of the segmented root network (Blaser et al., 2018) summarized in Figure 7.

There is a close, linear relationship (*R*^2^ = 0.991) between root length density *R*_*L*_ [cm/cm3] and root surface density [cm2/cm3], derived from the frequency of the smallest root distance (Figure 7a). This very good agreement is not surprising and only confirms the reliability of the complimentary approaches to estimate two highly correlated metrics. Another characteristic metric of the root distance histogram is the mean root-soil distance, i.e., its first central moment,

(5) $$\langle RDH\rangle =\sum_{i=1}^{d_{\max}}f_{i}d_{i}$$

with distance *d*_*i*_ and relative frequency *f*_*i*_. The relationship between *R*_*L*_ and mean root-soil distance 〈*RDH*〉 is non-linear (Figure 7b) with a huge reduction in mean distance by a relatively small *R*_*L*_ that is only composed of the tap root in the first week after planting. Note that 〈*RDH*〉 is bounded by *p* ≈ 3.5 cm, when the ROI is reduced to the maximum depth of the tap root (Figure 3b), as then *p* is simply the horizontal distance between the tap root and the ROI perimeter. The theoretical half mean distance *HMD* derived from *R*_*L*_ (Equation 1) and the measured mean root-soil distance 〈*RDH*〉 show good agreement (Figure 7b) which has already been reported previously (Koebernick et al., 2014). Note that *HMD* refers to root-root distances, whereas 〈*RDH*〉 refers to root-soil distances. The relationship between both entities depends on the spatial distribution of roots. This is shown by the following example.

**Figure 8:**
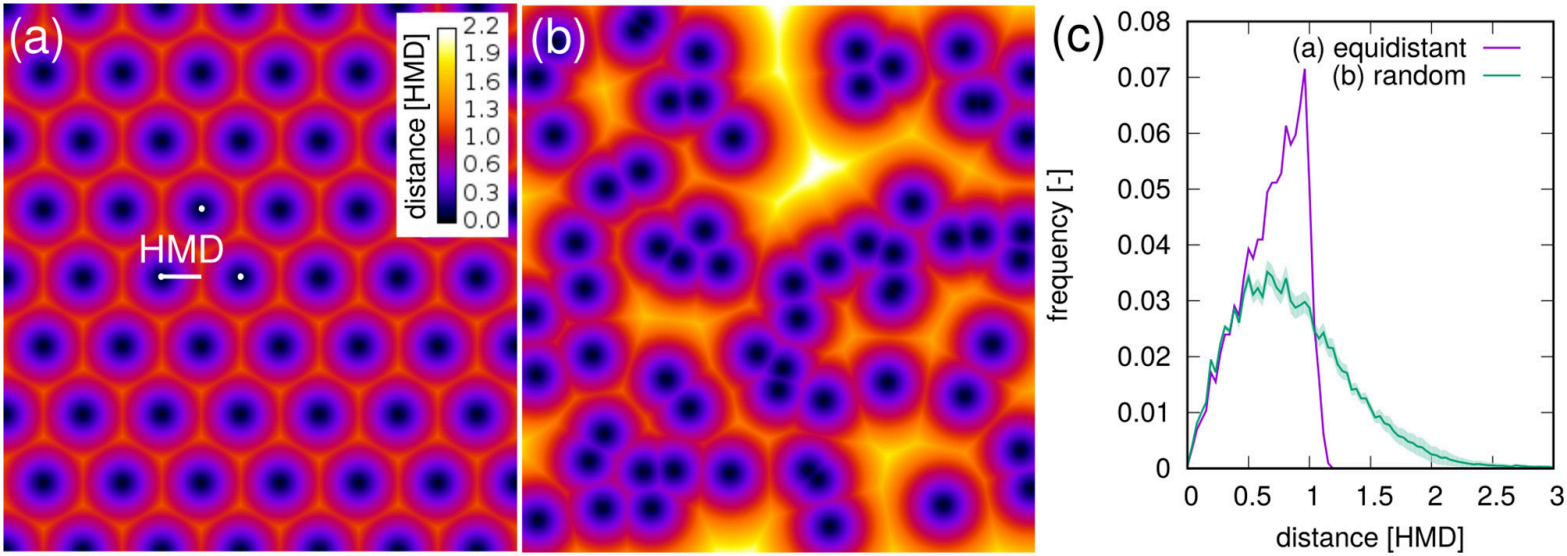
Root-soil distances in **(a)** an equidistant, hexagonal root bundle and **(b)** for a random pattern of roots in a two-dimensional cross section. The distances are normalized by the half-mean distance between roots. The root length density is the same in both point patterns. **(C)** The root distance histograms for both point patterns. Shaded areas for random point patterns represent standard deviation of ten realizations.

For a bundle of equidistant roots on a hexagonal lattice 〈*RDH*〉 amounts to 69% of the *HMD* (Figure 8a). For a random distribution of roots in two-dimensional cross sections it amounts to 89% of the theoretical *HMD* derived from *R*_*L*_ (Figure 8b). Evidently, the branching and clustering of roots changes the root distance histogram in characteristic ways (Figure 8c): (1) Short distances directly at the root surface (*d* < 0.5*HMD*) are just as abundant and mainly imprinted by the root length density itself. (2) Intermediate root distances (0.5*HMD* < *d* < *HMD*) are less frequent, when neighboring roots approach each other in the randomly distributed example (Figure 8b). That is, the local root length density increases by a factor of two, but not the volume fraction of intermediate distances between them. (3) Long distances beyond the equidistant spacing (*d* > *HMD*) can only occur in random point patterns. Taken together this leads to a higher 〈*RDH*〉 than what would be theoretically expected for a bundle for parallel roots with the same root length density. Note that these relations between root-root distances and root-soil distances are constant for a given pattern and do not depend on the actual spacing between roots (data not shown).

It turns out that for real root networks of *Vicia faba*, the 〈*RDH*〉 is in fact even larger than for random point patterns and amounts to 97% of the theoretical *HMD* derived from *R*_*L*_. This is also indicated by a 1:1 relationship in Figure 7c for all dates within 8-16 DAP, whereas the relationship starts to become non-linear and flattens out around 〈*RDH*〉≈2 cm or *R*_*L*_ < 0.08 cm/cm3 due to the incomplete exploration of the full ROI depth by the young tap root at 4 DAP. In a previous experiment with *Vicia faba* by Koebernick et al. (2014) the 〈*RDH*〉 amounted to a similar value of 93% of the theoretical *HMD* and the linear relationship between 〈*RDH*〉 and *HMD* started to flatten out around *R*_*L*_ < 0.12 cm/cm3 due to the same limitations in soil exploration by young roots. Real three-dimensional root networks differ from two-dimensional random point patterns in that they are continuous, i.e., they cannot emerge everywhere but have to branch and grow to explore the soil. This immanent alignment and clustering causes larger unexplored areas for the same root density in two-dimensional sections. As a consequence the normalized 〈*RDH*〉 is larger in real root networks of *Vicia faba* than in random root configurations. Future studies will show whether differences in root system architecture between different plant species lead to characteristic differences in the relationship between the introduced metrics for root length density and soil exploration.

The weighting factor *c* should be inversely related to the tap root fraction, which is confirmed by Figure 7e. There is some scatter in the data, which is presumably due to some correlation in the parameter set of the mixed triangular-gamma model leading to an equally good fit of the model to the RDH for a range of *c* values. Finally, the scaling parameter θ decreases as the root length density increases (Figure 7d). The relationship is mildly non-linear for 8–16 DAP, since an additional increase in root length density beyond 0.6 cm/cm^3^ does not lead to a proportional reduction in root distances for this root system architecture presumably due to the lack of secondary laterals. The large θ-values at 4 DAP are not reliable, since the weighting factor *c* is rather small, which renders the model fit insensitive to the θ-parameter.

In summary, the root distance traits reveal information that cannot be derived from conventional root system traits based on a skeleton analysis of the root network. The exact relationship between parameters derived from the root perspective (root network traits) and the soil perspective (root distance traits) hints to characteristic root growth patterns. An in-depth analysis of such scaling relations is out of scope of this study, as it would require a set of different plant species to compare different root system architectures.

### Strengths and limitations of soil perspective

The quantitative analysis of root growth patterns via root distance models has several advantages over traditional approaches based on root network analysis: (1) It is a more direct assessment of which soil volume is accessible to roots. (2) It can take into account variable extents of the rhizosphere with respect to different elements and processes (water uptake, nutrient uptake, rhizodeposits, etc.). (3) The proposed combination of root distance histograms and root age, obtained by differential imaging of registered X-ray CT datasets, enables a dedicated analysis of soil exploration by young roots. (4) Root distance analysis is more robust against image segmentation problems, as it is virtually insensitive to root surface roughness and small gaps, which are notorious problems for skeleton analysis of the root network.

Similar to dynamic root growth models based on root network traits (Leitner et al., 2010) the mixed triangular-gamma model proposed in the present paper lends itself to interpolation between sampling dates, since its parameters either change monotonically or remain rather constant. Especially during intermediate growth stages it might be necessary to carry out interpolation for different depths separately.

There are also some limitations of the description of root growth patterns from the soil perspective: (1) Branching angles, hierarchical ordering of laterals, length distributions of root segments and related traits of root networks cannot be assessed with root distance models. This is the reason why a combined analysis from the root perspective and the soil perspective may provide a more comprehensive representation of root growth patterns (2) Even though the mixed triangular-gamma model is versatile enough to model RDHs at all growth stages with only four parameters which have an easily conceivable, geometrical meaning, there is the downside that some parameters become unconstrained and start to fluctuate when the weighting factor of the corresponding model becomes too small or too large.

## Conclusions and outlook

We have introduced the mixed triangular-gamma model to describe root distance histograms, i.e., frequency distributions of Euclidean distances from soil to root, at several growth stages of *Vicia faba*. This new approach to assess root growth patterns from the soil perspective delivers complementary information to the traditional plant perspective based on root network analysis and facilitates a more direct assessment on rhizosphere processes.

In future work, the approach needs to be extended to further plant species, differing in root architecture. In particular the method needs to be tested for adventitious root architectures of grass species and in general for older plants. A prerequisite for such tests is obtaining 3D time resolved datasets with sufficient resolution to capture all roots, including fine roots. This is still a challenge for many grass species if the pot size is chosen to enable unrestricted root growth at least for the seedling stage. Another approach could be the extraction of undisturbed soil cores from the field, which enables the study of older plants with the trade-off of introducing field heterogeneity into the investigations. Finally, the approach can also be applied to root system architectures for a suite of plant species derived from dynamic root growth models like CRootBox (Schnepf et al., 2018). The benefits are two-fold. Metrics derived from root distance histograms may complement established skeleton-based metrics in high-throughput phenotyping. In addition, comparing parameter sets derived by model fitting to root distance histograms from plant species with vastly different root system architectures is helpful to scrutinize the physical meaning of each parameter in the proposed model.

## Author contributions

All authors conceived the project and wrote the manuscript. SS and MW carried out the analysis. SB and SS provided the underlying data.

### Conflict of interest statement

The authors declare that the research was conducted in the absence of any commercial or financial relationships that could be construed as a potential conflict of interest.
